# The rise of cochlear gene therapy

**DOI:** 10.1016/j.ymthe.2024.11.012

**Published:** 2024-11-08

**Authors:** Lukas D. Landegger, Ellen Reisinger, François Lallemend, Steffen R. Hage, Dirk Grimm, Christopher R. Cederroth

**Affiliations:** 1 Department of Otolaryngology, Vienna General Hospital, Medical University of Vienna, Vienna, Austria; 2 Department of Otolaryngology - Head and Neck Surgery, Stanford University School of Medicine, Palo Alto, CA, USA; 3 Gene Therapy for Hearing Impairment and Deafness, Tübingen Hearing Research Center, Department of Otolaryngology - Head and Neck Surgery, University of Tübingen, Tübingen, Germany; 4 Department of Neuroscience, Karolinska Institutet, Stockholm, Sweden; 5 Neurobiology of Social Communication, Tübingen Hearing Research Center, Department of Otolaryngology - Head and Neck Surgery, University of Tübingen, Medical Center, Tübingen, Germany; 6 Werner Reichardt Centre for Integrative Neuroscience, University of Tübingen, Tübingen, Germany; 7 Department of Infectious Diseases/Virology, Section Viral Vector Technologies, Medical Faculty and Faculty of Engineering Sciences, BioQuant Center BQ0030, University of Heidelberg, Heidelberg, Germany; 8 German Center for Infection Research (DZIF) and German Center for Cardiovascular Research (DZHK), partner site Heidelberg, Heidelberg, Germany; 9 Translational Hearing Research, Tübingen Hearing Research Center, Department of Otolaryngology - Head and Neck Surgery, University of Tübingen, Tübingen, Germany; 10 Department of Physiology and Pharmacology, Karolinska Institutet, Stockholm, Sweden

**Keywords:** cochlea, gene therapy, otoferlin, hearing loss, auditory, CSF, off-targeting, NHP

## Abstract

Recent evidence provides strong support for the safe and effective use of gene therapy in humans with hearing loss. By means of a single local injection of a set of adeno-associated virus (AAV) vectors, hearing was partially restored in several children with neurosensory nonsyndromic autosomal recessive deafness 9 (DFNB9), harboring variants in the *OTOF* gene. Current research focuses on refining endoscopic and transmastoid injection procedures to reduce risks of side effects, as emerging evidence suggests bidirectional fluid exchanges between the ear and the brain. Moreover, gene editing approaches and novel AAV capsids are successfully tested in animal models and will likely lead to enhanced targeting of the cochlea. Here, we cover the recent advances in cochlear gene therapy, provide an overview of the translational potential of these new approaches for existing and future clinical trials, and highlight the translational implications that remain to be determined for their application in humans.

## Graphical abstract

**Figure:**
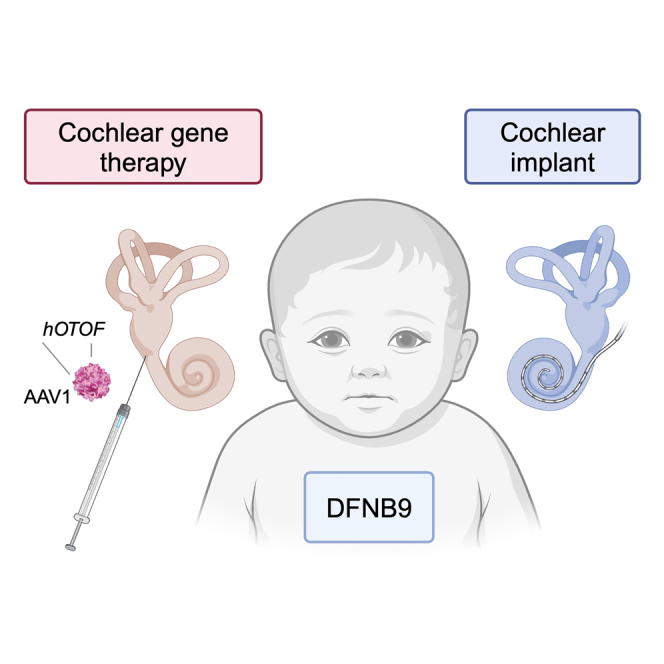

## Teaser

Landegger and colleagues discuss several recent clinical trials that have demonstrated gene therapy’s safety and effectiveness in treating hearing loss. While many questions remain, ongoing research aims to determine optimal injection procedures and develop new methods for better cochlear targeting, widening the spectrum of potential applications in humans.

## Introduction

Since the advanced engineering efforts in the 1970s by Clark, Hochmair, and Wilson, who were awarded the 2013 Lasker-DeBakey Clinical Medical Research Award, cochlear implants (CI) have been considered the major technology to restore hearing function in infants and adults with profound hearing loss.^1^ Nonetheless, the outcomes are unsatisfactory in nearly one out of five implanted individuals,^2^^,^^3^ and success in restoring speech comprehension declines even further with increasing age.^4^ Reasons for the broad and variable outcomes remain unclear but are thought to involve several device- and patient-specific factors such as signal processing strategies, intracochlear electrode location, operative approach, and genetics.^5^ Importantly, even in the best performers, the technical prosthesis never reaches the quality of natural hearing, resulting in reduced frequency resolution, difficulties in perceiving vocal emotions or recognizing speakers, and a higher cognitive load required for understanding speech.

In recent years, advances in the field of gene therapy have been made amenable to auditory investigation, with increasing pre-clinical research^6^ supporting the launch of several first-in-human trials currently recruiting in the US, Europe, and China (ChiCTR2200063181 and ClinicalTrials.gov: NCT05788536, NCT05821959, NCT05901480, and NCT06370351). These clinical trials target DFNB9 caused by variants in the gene *OTOF*, for which a dual-adeno-associated virus (dual-AAV) gene supplementation approach proved successful in neonatal mice.^7^^,^^8^^,^^9^ A pilot study in China revealed a successful gene therapy in two deaf children 5 and 8 years of age with *OTOF* variants, reaching near-normal hearing in the youngest child and partial restoration in the older child.^10^ Two months later, this study was followed by a publication in *The Lancet* of a Chinese trial confirming the benefits of *OTOF* gene therapy in five out of six deaf children with *OTOF* variants, with a recovery of hearing to thresholds between 38 and 50 dB persisting 26 weeks after surgery and major improvements in speech perception.^11^ Since the otoferlin cDNA is too long to be transported with a single AAV, whose cargo upper limit for coding sequences is 3.5–4 kb (together with regulatory elements such as promoters in a range of 1–1.5 kb), the strategy comprises splitting the cDNA for transport over two separate AAVs,^12^^,^^13^ as it was initially conceived for USH1B variants in *MYO7A.*^14^ The approaches have been tested in the pre-clinical setting and consist of pseudotyped AAV2/1 (in combination with a hair cell-specific promoter) or AAV2/Anc80L65, the reverse-engineered common ancestor of AAVs 1, 2, 8, and 9.^15^ Both capsid pseudotypes proved to transduce the inner hair cells, i.e., the sensory cells of the auditory system, in several different species.

## Main text

### Injection routes, how and why?

**Figure 1:**
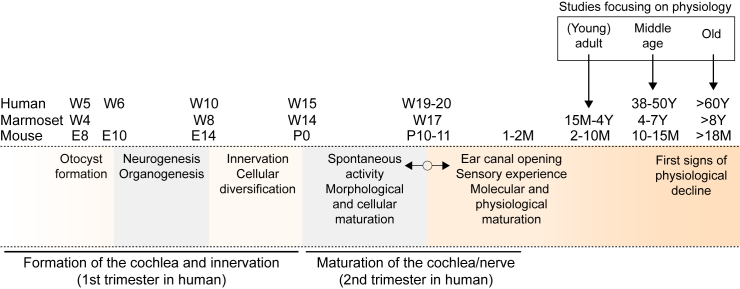
Comparative incongruence in the developmental stages of the mouse, marmoset, and human cochlea During the first trimester of gestation in humans occurs the formation of the cochlea and its innervation, starting with otocyst formation at week 5, neurogenesis and organogenesis between weeks 6 and 10, and the innervation with the accompanying cellular diversification until week 15. The development of the marmoset cochlea is almost parallelled to the human cochlea, anticipating it by nearly 1–2 weeks. The first trimester of cochlear development in humans corresponds to the whole gestational period in mice. The maturation of the cochlea and the innervation occur during the second trimester in humans, corresponding to the postnatal period in the mouse. This is when spontaneous activity emerges, allowing the refinement of the innovation together with the morphological maturation of the cochlea. The ear canal opens, and the first sensory experiences are perceived. In mice, the cochlea is considered molecularly and physiologically mature between 1 and 2 months of ages, which corresponds to 15 months of age in marmosets and the end of the second trimester in humans. The first signs of physiological decline occur around middle age (10–15 months of age in mice, 4–7 years in marmosets, 38–50 years of age in humans), with more profound hearing symptoms at older age (>18 months in mice, >8 years in marmosets, >60 years in humans) due, at least in part, to hair cell dysfunction, neural loss, and strial atrophy. E, embryonic day; P, post-natal day; W, week; M, month; Y, year.

**Figure 2:**
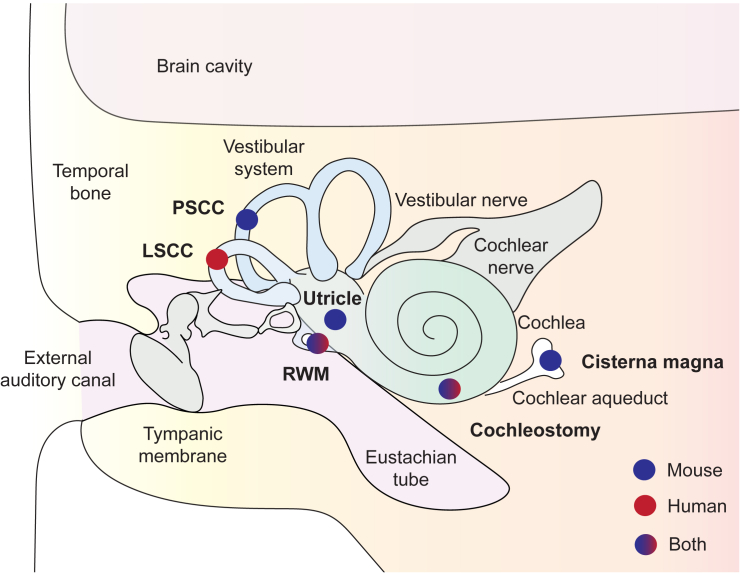
Gene delivery routes to the cochlea Illustration of local administration routes for gene therapy including posterior semicircular canal (PSCC) in mice (blue dots) or lateral semicircular canal (LSCC) in humans (red dots), injections into the utricular cavity and cochleostomy (in mice), and transmastoid or transtypmanic round window membrane (RWM) injections that are performed on both species (blue/red gradient dots). Cochleostomy is used for cochlear implantation in humans in the event of complicated anatomy and difficult access to the RWM, and as not been yet used as a delivery route for gene therapy in humans. Injections in the cisterna magna (CM) in mice lead to direct access to cerebrospinal fluid (CSF) transportation toward the subarachnoid space and into the scala tympani of the cochlea via the cochlear aqueduct. While this injection is systemic, it allows for bilateral transduction with a single injection. While gene therapy for hearing disorders via the CSF has not been yet performed, this surgical intervention may become a possible route owing the systemic safety of the selected viral vectors.

Historically, gene delivery in pre-clinical models (primarily rodents) has been performed in newborn animals (before hearing onset, which occurs in mice near postnatal day [P]12–P14),^6^ as the cochlea is more accessible and less surgical trauma is required compared to targeting the bony and dense structure of the adult and mature cochlea. Only recently has the delivery of gene therapy also been successful in adolescent^8^ or adult mice,^9^^,^^16^ which has more translational relevance to human newborns or children, where the cochlea is already mature (hearing onset in a human fetus is at week 19–20).^17^ Figure 1 shows the comparative developmental timescale between the human, marmoset, and mouse cochlea. Local administration has historically been a preferred injection route, mainly because the inner ear was considered to be “immune-privileged” due to the existence of the blood-labyrinth barrier. In addition, local injection into the inner ear fluids was assumed to permit a higher concentration of therapeutic vectors concurrent with a lower risk of side effects due to spreading to the body when compared to systemic injections. Local delivery to the cochlea mainly involves four types of surgical approaches,^18^^,^^19^ all of which have advantages and disadvantages^20^^,^^21^: (1) injections via the posterior semicircular canal (PSCC), (2), injection into the utricular cavity, (3) round window membrane (RWM) injections, and (4) more invasive strategies such as cochleostomy (Figure 2).^22^^,^^23^ When used alone, each of these methods comes with adverse effects that complicate direct translation. Injections via vestibular organs (1 and 2) may be hampered by the deep location of these structures in the human temporal bone and may entail additional risks of vestibular dysfunction. Cochleostomy inevitably causes damage to inner ear structures such as the stria vascularis, resulting in irreversible hearing loss in animal models. In humans, this approach represents one of the most established procedures to introduce a CI. Injection through the RWM without any fenestration of the bony shelf of the inner ear results in an overpressure, which is most likely released via the cochlear aqueduct (CA, *aquaeductus cochleae*).^20^^,^^21^ This fluid pressure causes an escape of viral vectors toward brain structures, plausibly via the cerebrospinal fluid (CSF), and may be the cause for the lower transduction rates of basal versus apical hair cells observed not only in rodents but also in non-human primates (NHPs).^24^ In contrast, RWM injection in combination with fenestration, e.g., in the semicircular canals,^24^^,^^25^ allows for a better release of pressure and thus a better distribution of the viral vectors along the whole cochlea. After extensive pre-clinical studies, some of the above-mentioned human trials refined their respective delivery methods by either (1) creating a small hole in the lateral semicircular canal or (2) introducing a fenestration of the oval window to allow overpressure release of the inner ear fluids during RWM injection.^24^ Another difference is that most trials favor a minimally invasive transcanal (i.e., through the external auditory canal) endoscopic procedure, which has proved particularly useful for ear surgery in the pediatric patient population, while others rely on the typical transmastoid procedure known from CI surgery.^26^

### Bidirectional fluid connections between the brain and the cochlea

Intriguingly, for all the above-mentioned injection methods, a transduction of the contralateral, non-injected ear has been observed in rodents,^21^^,^^27^^,^^28^^,^^29^ pointing toward a fluid bridge that allows the transfer of viral vectors from one ear to the other. Whether the transduction of these potent AAVs in other tissues outside the inner ear and their passage through the CA towards the CSF also occurs in NHPs and humans is under debate. For instance, despite widespread transduction of the murine cerebellum after RWM injection,^29^ genome copy levels of AAV2/Anc80L65 (and AAV2/1) were around background level in the central nervous system of rhesus monkeys (*Macaca mulatta*) using a similar administration route.^24^ In a recent study in rhesus monkeys and vervet monkeys (*Chorocebus aethiops*), CSF delivery of three alternative AAV pseudotypes (AAV2/1, AAV2/2, and AAV2/9) via intracerebroventricular injection also resulted in cochlear transduction,^30^ suggesting that brain and cochlear fluids also communicate in the NHP.

The CA, connecting the perilymph to the CSF, was suggested to underlie this phenomenon. Indeed, recent pre-clinical research has evidenced the connection of the CSF toward the ears’ perilymph using a combination of *in vivo* time-lapse magnetic resonance imaging with a 9.4 T pre-clinical scanner, computed tomography, and optical fluorescence microscopy.^31^ The contrast agent gadobutrol reached the cochlea via the CA within a minute after its injection in the CSF-filled cisterna magna (CM). Concerning AAVs, two independent studies in mice demonstrated that a single injection of AAV vectors into the CM can bilaterally transduce the cochlea’s sensory inner hair cells^31^^,^^32^ and even rescue hearing in an adult mouse model of DFNA25, an autosomal dominant form of progressive, nonsyndromic deafness.^31^ While the fluid passage from the cochlear perilymph toward the CSF has not been evidenced with neuroimaging, the cumulative evidence suggests that there is a bidirectional fluid exchange happening between the cochlea and the CSF.

Such fluid connection between the CSF and the cochlea has also been evidenced in humans. Cisternograms, which are used in individuals to assess CSF leaks at the base of the skull, have shown a progressive diffusion of gadolinium contrast into the human cochleae and vestibule in 15 patients.^33^ In humans, the size of the CA is close to that of the rodent (100 μm wide and 400 μm long), making it relatively small with respect to the overall anatomic size. Its patency is thought to decline with age,^34^^,^^35^ albeit histological artifacts from human temporal bone cannot be discarded. Other studies have inferred the patency of the CA by means of intratympanic pressure changes resulting from intracranial or postural changes,^36^^,^^37^^,^^38^^,^^39^^,^^40^ suggesting that the human CA is functionally patent in 89% of young adults and 70% of older adults. Recently, high-resolution temporal bone computed tomography revealed that the CA is undetectable in 14 (3/22) of adults, whereas this only occurred in 1% of children (1/85).^41^ Additional studies are needed in humans to better understand the CA patency and fluid exchanges occurring between the cochlea and the brain. Nonetheless, these findings demonstrate that fluids from the brain are connected to those of the cochlea.

### Tissue off-targeting and side effects

Few studies have investigated the potential tissue off-targeting effects and negative effects of gene delivery in the ear. The study from Mathiesen et al., who injected an AAV9-PHP.B vector in the CM in mice, is a proper illustration of such analysis, including the assessment of virus expression, changes in immune responses, and alterations in behavior. In the context of CM injections, no transduction in the liver, cortex, or CA3 region of the hippocampus was found.^31^ However, tissue off-targeting was identified in <5% of mature neurons expressing the neuronal nuclear antigen (NeuN+) marker in the CA2 region of the hippocampus and <20% in the cerebellum. Although there was no evidence of cochlear or brain inflammation and no impact on mouse behavior, these findings suggest that the CSF route has non-negligible risks of targeting other cells and tissues than the cochlea. Several clinical trials are using intracisternal delivery of AAVs for neurodegenerative diseases (ClinicalTrials.gov: NCT03580083, NCT03566043, NCT04127578, NCT04408625, NCT04411654, and NCT04713475), and pioneering experiments in humans have been performed without major complications,^42^^,^^43^ Conversely, a recently completed trial (ClinicalTrials.gov: NCT02362438) using intrathecal gene therapy against giant axonal neuropathy revealed several adverse effects, one of them being serious (possibly linked to the treatment itself rather than the surgery).^44^ While postmortem analysis of inner ear transduction (which would be an off-target tissue in these studies) may reveal the potential for bilateral cochlear targeting when considering intrathecal delivery, the existing evidence emphasizes the need for safety assessments of the vectors derived from pre-clinical research, whether performing local injections or intrathecal.

### Mitigation of off-targeting effects with vector and genetic bioengineering

As AAV vectors lack inherent selectivity for specific cell populations, there is a need to enhance targeting strategies to minimize tissue off-targeting and side effects. In this regard, recent advances in technologies facilitating transcriptomic and epigenetic analysis of tissues at the single-cell level offer the potential to identify regulatory elements capable of confining viral expression to distinct tissue and cell types.^45^ Similarly, as our knowledge on cell-type-specific cell-surface receptors in the ear increases,^46^ this will enable novel approaches for targeting at the transduction level, for instance, by displaying bispecific DARPins (Designed Ankyrin Repeat Proteins) on the AAV capsid.^47^ Accordingly, with optimal bioengineering of AAV capsids and the selection of promoters specific for the respective cochlear target cells, the benefits should ultimately outweigh possible harms.

Another approach to mitigate side effects could involve the use of AAV vectors to deliver CRISPR-based genome editing components to specifically target hearing-related genes. One can assume that rectifying a mutant transcript with the CRISPR-based gene correction method would theoretically only affect the cell types that express it, thereby potentially enhancing cellular specificity. CRISPR-Cas editing has demonstrated efficient results for treating autosomal dominant disorders caused by mutated genes,^48^^,^^49^ including hereditary deafness.^50^^,^^51^^,^^52^ Importantly, the versatility of the CRISPR-based tools extends to a wide range of applications, including DNA and RNA editing, as well as modulating gene expression that could be unique to the cochlea. However, the CRISPR-Cas technology may also display genetic off-targeting and act on non-specific genomic regions. In this regard, several variants of Cas9 have been developed,^53^^,^^54^ with a decreased likelihood of genetic off-targeting and limited impact on efficiency. Furthermore, given the potential of CRISPR gene therapies to address human diseases, it is anticipated that AAV vectors combined with Cas9 variants would deliver heightened efficiency with minimal genetic off-targeting in the near future.

The development and refinement of CRISPR and AAV reagents to yield a broader yet more precisely targeted array of molecular therapeutics holds great promise. Achieving this goal requires a convergence of multidisciplinary expertise spanning fields such as microbiology, molecular biology, computational and systems neuroscience, physiology, clinical knowledge, and translational methodologies. This collaborative effort will be instrumental in unlocking the potential of these technologies for transformative therapeutic applications. Until then, these novel research findings are exciting for the field and will hopefully contribute to the optimization of inner ear gene therapy applications as well as eventually the treatment of patients with precision medicine.

### Translational validation and potential of the NHP models

While the efficacy of gene therapy approaches for restoring hearing is investigated in knockout mouse models, which phenocopy the human hearing phenotype with relatively high fidelity, the cellular specificity of AAV vectors as well as their safety are best assessed in large animals and particularly NHP models. The rhesus monkey has been a preferred model, with established standard operating procedures for the assessment of hearing function using auditory brainstem responses and (distortion product) otoacoustic emissions.^55^ The AAV2/9-PHP.B vector has already been used in a variety of animal studies^56^ and is among the rare vectors applied to the rhesus monkey inner ear, together with AAV2/S,^57^ AAV2/1,^58^ and AAV2/Anc80L65.^24^ However, no behavioral data exist at the moment, and other NHP models such as the marmoset monkey (*Callithrix jacchus*) may have to be considered for securing the translation of the rodent findings toward the bedside. Marmosets, smaller in size, are an established NHP model for auditory research,^59^ whose anatomical features of the cochlea are similar to that of humans.^60^^,^^61^ Marmosets are highly vocal animals that exhibit complex vocal behavior with several features that can also be found in human speech^62^ and with a larger call repertoire and more frequent vocalizations than what is found in the rhesus monkey. Consequently, this model could allow an assessment of hearing gene therapy not only at the neurophysiological level but also at the behavioral level.^63^ One example is the fact that the maturation of marmoset vocalizations from infancy toward adulthood^64^ requires parental feedback.^65^ Consequently, marmosets that have limited parental interactions maintain immature vocalizations.^66^ Using marmosets could thus help determine whether the improvement in hearing is sufficient for the marmosets to fully recover their species-specific vocal behavior, which is dependent on the proper auditory perception of the vocal signals from their parents. Additionally, marmosets engage in turn-taking behavior, communicating with each other in a predictable manner,^67^^,^^68^ and use their vocalization to individually label conspecifics.^69^ Furthermore, the vocal behavior of adult marmosets is dependent on auditory feedback^70^ and can be modulated by auditory perturbation.^71^^,^^72^ Finally, marmosets could be trained to flexibly detect and discriminate sounds.^73^ These features of marmoset vocalization and hearing allow us to quantitatively investigate auditory performance in controlled experimental designs.

## Conclusions

Delivery is still the major obstacle hindering *in vivo* gene therapy, and overcoming this challenge is pivotal. At least in the hands of an experienced surgeon who knows how to avoid the facial nerve, the worst complication of intracochlear delivery of a novel compound would likely be a deaf ear. Is it reasonable to replace this with the risk of accessing the intracranial space as well as the need to administer larger volumes and quantities of virus, even if both inner ears could be reached at the same time? In the event that a low level of tissue off-targeting expression of the transgene is achieved and transduction rates in the on-target tissue are comparable, the injection route that is the least invasive (whether local or systemic) for the patient should be chosen. As the CSF route appears to be relatively atraumatic to the ear, and as it might require only one surgery for transducing both ears, with more evidence of its impact on the brain and body, it could become an accepted therapeutic route for bilateral deafness in the future.

Should the final results from the *OTOF* trial provide compelling evidence of efficacy and limited toxicity, the new 2023 FDA Modernization Act 2.0—which favors the use of non-animal methods as alternatives for drug safety and efficacy testing^74^^,^^75^—would propel the development of inner ear gene therapies that could reach the bedside, at least in the US. However, while there are outstanding developments of human-based *in vitro* alternatives (e.g., organoids) for a large number of organs,^76^ the hearing field is at its infancy and deserves greater attention.^77^ Until the translational validity of these non-animal-based methods is established, for the purpose of testing both efficacy and safety, the complex cellular architecture of the inner ear^78^ will require the use of animal models for testing gene therapy.

Should all these questions be successfully and positively addressed, will this be the beginning of the end of a CI era? Likely not. Because the majority of genes whose variants cause hearing loss are required during the development of the cochlea, a postnatal supplementation via viral vectors will not restore hearing in humans.^17^ In addition, while monogenic factors largely account for more than half of the cases of congenital hearing loss in developed countries,^79^ hearing loss in adults is mainly driven by environmental factors (e.g., noise exposure or ototoxic medication) accompanied by monogenic defects caused by ultrarare variants with a range of onset extending well into advanced age^80^ as well as polygenicity.^81^ Accordingly, for these patients, such targeted therapeutics would not be a viable solution. Rather, we foresee a future where gene therapy and CI technologies may join forces toward optimizing personalized medicine in otolaryngology.

## Acknowledgments

L.D.L. is supported by a Career Development Award from the American Society of Gene & Cell Therapy and the 10.13039/100001545Children's Tumor Foundation. The content is solely the responsibility of the author and does not necessarily represent the official views of the American Society of Gene & Cell Therapy. The financial support of the Austrian Federal Ministry for Labour and Economy and the 10.13039/100010132National Foundation for Research, Technology and Development, and the 10.13039/501100006012Christian Doppler Research Association is gratefully acknowledged. E.R. receives funding from the 10.13039/501100001659German Research Foundation (DFG) for support of this work via the Heisenberg program (#416097726) and a research grant (#416116807). C.R.C. receives funding from the 10.13039/501100004359Swedish Research Council (Vetenskapsrådet #2023-02326) and the 10.13039/100016608Rainwater Charitable Foundation.

## Author contributions

L.D.L., E.R., F.L., S.R.H., D.G., and C.R.C. played a role in the conceptualization, writing, and funding acquisition for the writing the manuscript and approved the final version.

## Declaration of interests

L.D.L. has received research funding from Decibel Therapeutics/Regeneron Pharmaceuticals and Amgen and has worked as an independent consultant for Conclave Capital and Gerson Lehrman Group. E.R. is co-inventor on a patent application for dual-AAV vectors to restore hearing. The University Medical Center Göttingen has licensed the rights to these parts of the patent exclusively to Akouos, Inc., USA. C.R.C. has a pending patent application for the delivery of therapeutics to the inner ear via the cisterna magna (international patent application no. PCT/US22/82225, title: “CSF transport pathway for delivery of agents to the inner ear”).
